# Does pathway analysis make it easier for common variants to tag rare ones?

**DOI:** 10.1186/1753-6561-5-S9-S90

**Published:** 2011-11-29

**Authors:** Hae-Won Uh, Roula Tsonaka, Jeanine J Houwing-Duistermaat

**Affiliations:** 1 Department of Medical Statistics and Bioinformatics, Leiden University Medical Center, Einthovenweg 20, 2333 ZC Leiden, The Netherlands; 2 Netherlands Consortium for Healthy Ageing, Leiden University Medical Center, P.O. Box 9600, 2300 RC Leiden, The Netherlands

## Abstract

Analyzing sequencing data is difficult because of the low frequency of rare variants, which may result in low power to detect associations. We consider pathway analysis to detect multiple common and rare variants jointly and to investigate whether analysis at the pathway level provides an alternative strategy for identifying susceptibility genes. Available pathway analysis methods for data from genome-wide association studies might not be efficient because these methods are designed to detect common variants. Here, we investigate the performance of several existing pathway analysis methods for sequencing data. In particular, we consider the global test, which does not consider linkage disequilibrium between the variants in a gene. We improve the performance of the global test by assigning larger weights to rare variants, as proposed in the weighted-sum approach. Our conclusion is that straightforward application of pathway analysis is not satisfactory; hence, when common and rare variants are jointly analyzed, larger weights should be assigned to rare variants.

## Background

Genome-wide association studies (GWAS) have found hundreds of single-nucleotide polymorphisms (SNPs) that are significantly associated with several complex traits. In most cases, however, these SNPs explain only a small proportion of the known genetic variance. A possible explanation is that causal variants have a lower minor allele frequency (MAF) than common SNPs. Moreover, rare variants are weakly correlated with common SNPs, because MAFs must be similar for two variants to be highly correlated [1]. It follows that common variants may have only a limited capacity to tag rare variants. This limitation in GWAS, in which common variants with a frequency of 5% or higher were used, leads to a direct search for associations with multiple rare variants. Recently, efforts have been made to genotype rare variants, such as in the 1000 Genomes Project (http://www.1000genomes.org). The low frequency of rare variants, however, may result in low power to detect associations. Given currently available sample sizes, there is a need for powerful methods to detect association with multiple rare variants. We investigate whether pooling variants by pathway into a composite test provides an alternative strategy for identifying susceptibility genes.

Pathway analysis methods have been developed in the context of gene expression data analysis. There are two general approaches: competitive and self-contained. The distinction lies in the definition of the null hypothesis and in the calculation of the *P*-value [2,3]. Concerning the definition of the null hypothesis, a competitive test compares differential expression of the gene set to a standard defined by the complement of that gene set. In contrast, a self-contained test compares the gene set to a fixed standard that does not depend on the measurements of genes outside the gene set. Concerning the calculation of the *P*-value, the competitive test bases the calculation of the *P*-value for the gene set on a distribution in which the gene is the sampling unit, whereas the self-contained test takes the subject as the sampling unit.

Recently, these methods have been extended to data from GWAS to detect pathway association. Compared to expression data, several distinctive features in the data from GWAS require different methodological considerations. In contrast to gene expression data, in which each gene contains one expression value, in data from GWAS each gene contains many SNPs that are in linkage disequilibrium (LD). Here lies the distinction between one- and two-stage approaches; the first approach ignores these biological features, and hence the second approach might be more powerful [4]. Incorporation of LD information, however, might be less essential if we focus on rare variants using sequencing data because of weak correlation of rare variants with common SNPs.

In this paper, using the data provided by Genetic Analysis Workshop 17 (GAW17) [5] and several pathway analysis methods, we test the vascular endothelial growth factor (VEGF) pathway that influences quantitative trait Q1. Because we have a target gene set (we knew the “answers” to the GAW17 simulation), the natural choice is the self-contained methods that consider only the genes or SNPs involved in the specific gene set. The first method to be applied is the global test (GT) [6]; this test was designed for gene expression data and uses all SNPs without consideration of gene-level effects (one-stage approach). Next we consider two two-stage approaches that were designed for detecting common variants: the gene set ridge regression in association studies (GRASS) algorithm [4] and a new test called the empirical Bayes (EB) method [7]. At the gene-level, the GRASS algorithm uses LD information within a gene using principal components analysis, and at the pathway level the GRASS algorithm uses ridge regression. The EB method uses a mixed-effects model with a general random effects structure at the pathway level and uses the empirical Bayes estimates of the random effects of the first stage as covariates in the model for the phenotype. Because the GRASS algorithm can deal with only binary outcome, the GAW17 affected-unaffected phenotype serves as the outcome variable for these analyses.

The methods we use were designed to detect multiple common variants with small effects. To better deal with the low frequency of rare variants, we propose to weight counts of each rare variant (MAF < 1%) on the basis of the estimated variance under the null hypothesis of no association. We propose the weighted global test (WGT), in which the weights are defined as in the weighted-sum method [8] for rare variants and take a value of 1 for common variants in the GT.

Once significant pathways are identified, we might want to pinpoint the associated genes for further analysis. We consider three methods at the gene level: the combined multivariate and collapsing (CMC) method [9], the weighted-sum approach [8] that analyzes only rare variants, and the WGT. For details of the first two methods, we refer to Dering et al. [10].

All programming has been conducted in R (http://www.r-project.org). The R package globaltest can be obtained at http://www.bioconductor.org, and the source code for GRASS (SNPath) can be downloaded at http://linchen.fhcrc.org/grass.html.

## Methods

### Study sample

We consider data from 697 unrelated individuals. We analyze the 125 SNPs (28 common and 97 rare SNPs) in 9 genes from the VEGF pathway, which influences quantitative risk factor Q1. The MAFs range from 0.07% to 16.5%. The dichotomous phenotype Affected serves as the outcome variable (488 control subjects and 209 case subjects). We apply the pathway analysis methods to the 200 simulated GAW17 data sets to study the power.

### Pathway analysis methods

We begin by establishing some notation. For *N* individuals at *J* loci, we let *x_i_* = (*x_i_*_1_, …, *x_iJ_*)′ be a column vector in which *x_ij_* is the genotype at locus *j* in individual *i*. We code each genotype as 0, 1, or 2, corresponding to the number of minor alleles present at that locus. We also let *Y_i_* denote the disease status of individual *i*, which is coded 0 for control subjects and 1 for case subjects. We then let *X* = (*X*_1_, …, *X_N_*)′ be an *N* × *J* matrix of genotype data and let *Y* = (*Y*_1_, …, *Y_N_*)′ be the *N* × 1 vector of disease status. In addition, for two-stage approaches, we denote *g* = 1, …, *G* for *G* genes in a pathway.

The GT is a one-stage approach and is based on a regression model that predicts response from the SNPs of a gene set. We use generalized linear models to model the dependency of response *Y* on gene expression measurements *X* of a set of *J* SNPs on *N* samples:(1)

where *h* denotes the link function. Under an assumption that all regression coefficients are sampled from a common distribution with mean 0 and variance *τ*^2^, instead of testing *H*_0_: *β* = (*β*_1_, …, *β_J_*) = 0, one can test a new null hypothesis: *H*_0_: *τ*^2^ = 0. Using the notation:(2)

the model simplifies to a random effects model:(3)

The null hypothesis can then be tested, based on a score test statistic [11,12]:(4)

where *R* = (1/*m*)*XX*′, *μ* = *h*^−1^(*α*), and *μ*_2_ is the second central moment of *Y* under the null hypothesis . It can be shown that *Q* is asymptotically normally distributed. However, the *p*-value is calculated based on permutations of samples (case and control labels).

For the weighted version of the GT, the WGT, the weights *w* = (*w*_1_, …, *w_J_*)′ are added to the model as follows:(5)

We assign more weight to rare variants (MAF < 1%), as calculated in the weighted-sum approach, as follows [8]. Let *n_j_* be the total number of subjects genotyped for variant *j*,  be the number of mutant alleles observed in the control subjects, and  be the total number of individuals genotyped for variant *j*. The weight:(6)

is the expected variance based on allele frequencies *q_j_* computed from control subjects only:(7)

We assign the weight 1 to common SNPs.

The GRASS algorithm is a two-stage approach. At the gene level the GRASS algorithm captures LD structure with principal components analysis. For each gene, it selects all nontrivial eigen-SNPs that explain about 95% of the gene variation, and a least absolute shrinkage and selection operator (LASSO) regression is used to summarize the most representative eigen-SNPs:(8)

for *k* ≤ *J* eigen-SNPs. At the pathway level, the group ridge regression is applied, and the statistic can be written:(9)

The significance of the statistic is determined by permutation of case and control labels.

The EB method is a two-stage approach. In the first step we summarize the subject-specific SNP information using the EB estimates of the random effects of a mixed-effects logistic regression. In the second stage, the obtained EB estimates are used as covariates in the phenotypic model to test for association of genes. For details we refer to Houwing-Duistermaat et al. [7]. Let *X_igj_* be the genotype at locus *j* in gene *g* for individual *i*. Let *b_i_* be the random effect for subject *i*, and let *b_ig_* be the random effect of gene *g* of individual *i*. Under Hardy-Weinberg equilibrium, *X_igj_* given *b_i_* and *b_ig_* is assumed to follow a binomial distribution with the number of trials being 2 and a probability *π_igj_*. The probability is modeled as follows:(10)

For each gene the EB estimate is given by:(11)

Intuitively, the EB estimate can be interpreted as follows: The more minor alleles an individual has over all the SNPs in a gene, the higher the EB estimate will be for this gene.

### Calculation of type I error rate and power

Because none of the methods have been applied to pathway analysis with multiple common and rare variants, we investigate the type I error rate and power of each method. To determine the type I error rate, we simulate phenotype under the null hypothesis of no association; we generate 1,000 samples from the normal distribution and dichotomize the phenotypes by taking the top 30% as case subjects and the rest as control subjects. The type I error rates of the methods are then calculated as the proportion of significant replicates at the nominal level of 5% out of 1,000 replicates. For the type I error rate, 95% of the empirical estimates of 1,000 replicates are expected to fall between 0.036 and 0.064. The empirical power is determined by calculating the number of times the *P*-values are smaller than the preset threshold of 0.05 divided by 200, the number of simulated phenotypes.

## Results

We first compared four pathway analysis methods: GT, GRASS, the EB method, and WGT. These tests were performed for 697 unrelated individuals, and the *P*-values were obtained by analyzing functional and nonfunctional SNPs (28 common SNPs and 97 rare SNPs in 9 genes). As shown in Table 1, type I error rates for all methods were at the 5% level. The powers of the GT (41%) and GRASS algorithm (43%) were comparable, but the EB method had little power (5%) to establish association. The WGT, in which rare variants received more weight, as described in the Methods section, had an increased power of 65%.

**Table 1:** Type I error rate and empirical power of tests for 125 SNPs in the VEGF pathway

Method	Type I error rate	Empirical power
Global test	0.044	0.41
GRASS	0.047	0.43
Empirical Bayes	0.055	0.05
Weighted global test	0.041	0.65

To narrow down the candidate region and possibly pinpoint the associated genes for further analysis, we conducted an association analysis of each gene in the pathway. Because the WGT performed best at the pathway level, we compared the WGT to the CMC and weighted-sum methods. In Table 2, the power of the three methods at each gene is given. Nine genes influence the Q1 quantitative risk factor, but *VEGFC*, containing only one rare SNP, was not analyzed. Except for *FLT1*, the power of all three methods was low, and the CMC method outperformed the other methods. For *FLT1*, for which more rare variants were included, the two methods that assign more weight to rare variants performed better.

**Table 2:** Empirical power of tests for genes in the VEGF pathway

Gene	Number of SNPs			Empirical power		
	
	Total	Common	Rare	CMC	Weighted-sum	Weighted global test
*ARNT*	18 (5)	3 (1)	15 (4)	0.31	0.28	0.12
*ELAVL4*	10 (2)	3 (0)	7 (2)	0.27	0.10	0.14
*FLT1*	35 (11)	10 (3)	25 (8)	0.80	0.83	0.84
*FLT4*	10 (2)	2 (0)	8 (2)	0.03	0.02	0.02
*HIF1A*	8 (4)	1 (1)	7 (3)	0.03	0.02	0.03
*HIF3A*	21 (3)	6 (0)	15 (3)	0.13	0.04	0.15
*KDR*	16 (10)	2 (2)	14 (8)	0.16	0.09	0.08
*VEGFA*	6 (1)	1 (0)	5 (1)	0.07	0.05	0.08
*VEGFC*	1 (1)	0 (0)	1 (1)			

The number of functional SNPs is shown in parentheses.

**Figure 1:**
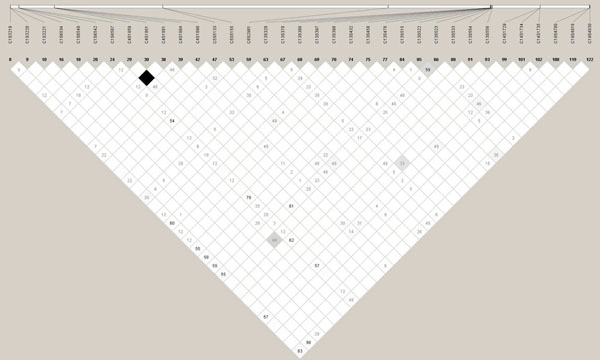
**Linkage disequilibrium of 125 SNPs using *r*^2^ in the VEGF pathway.** The low *r*^2^ ≈ 0 values (white cells) show weak correlations between the SNPs. The white cells indicate absence of LD (*r*^2^ = 0), shades of grey intermediate degree of LD (0 <*r*^2^ < 1), and black cells high LD (*r*^2^ = 1).

**Figure 2:**
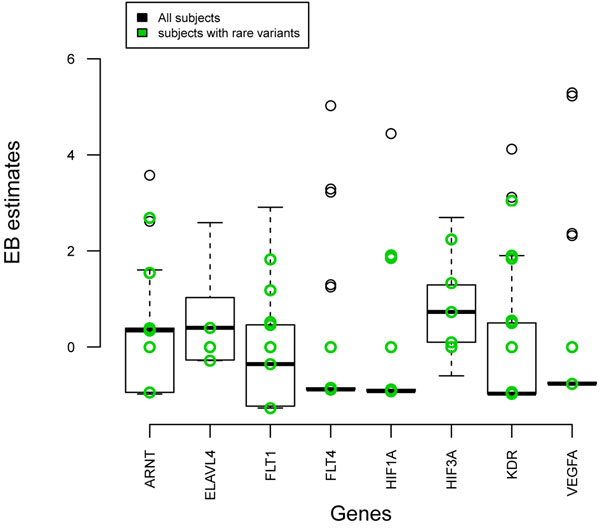
**Empirical Bayes estimates of individuals per gene**. The EB estimates were obtained using the EB approach in a gene-level analysis and summarize the amount of information contained by each gene. Higher EB estimates indicate more information for the gene. The green circles depict individuals with rare variants.

Next we investigated whether the common variants might tag rare variants. We first examined the correlation between the SNPs. We used Haploview [13] to produce a LD plot using the *r*^2^ measure. Figure 1 shows weak correlations (*r*^2^ ≈ 0) between the 125 SNPs. Then, we examined the EB estimates, which reflect the summarized information per gene, for all individuals (Figure 2). Overall, there was little evidence that common variants tagged rare ones. Otherwise, the EB estimates of the subjects with rare variants (green circles) would have had higher EB values to contribute to overall statistics. For *FLT1* and *KDR* the contribution of subjects with rare variants was comparable to the contribution of subjects with common variants.

## Discussion and conclusions

We investigated the performance of several pathway analysis methods to detect multiple rare variants that are associated with disease. The empirical power for all methods was low. The powers of the GT and GRASS algorithm were comparable, even though the GT did not use gene-level information (such as LD in a gene and the size of genes). As depicted in Figure 1, rare and common SNPs were weakly correlated with each other. Adding LD information, therefore, would not improve efficiency. The GRASS algorithm uses principal components analysis at the gene level, and the robustness of the principal components analysis for summarizing rare variants should be investigated. By giving more weight to rare variants, the power of the WGT increased from 41% to 65%. Improvement by using a variable threshold for rare variants or other methods might be possible. The power of the EB method was poor, which implied that the EB method cannot deal with rare variants well. This was also confirmed by investigating the EB estimates of individuals with rare variants (Figure 2). A larger number of common and rare variants within a gene might improve the EB approach. Improvements might also be possible by better summarizing the information on rare variants at the gene level by collapsing rare variants and including family data.

We also studied the type I error rate by using (200) dichotomized Q4 phenotypes, because Q4 phenotypes are assumed not to be associated with any SNP in the VEGF pathway that influences Q1. This resulted in extremely inflated type I error rates. To investigate possible correlation between dichotomized phenotypes Q4 and the Affected status, we constructed a simple 2 × 2 table. The concordance rates for 200 data sets were 68.2–75.9% with an average of 72.5%; that is, 72.5% of subjects had the same case-control status in both phenotypes. Simulating phenotype under the null hypothesis of no association seems a better practice for estimating type I error rate.

Concerning the conjecture of whether pathway analysis would be helpful for detecting multiple common and rare variants jointly, both Figures 1 and 2 show little evidence of common variants tagging rare ones. In Figure 1 we used the *r*^2^ measure. If both of two loci have rare alleles, it is possible for *D*′ to be 1 and *r*^2^ to be small. Hence, *D*′ is hard to interpret with rare alleles, and we chose to use *r*^2^, which is a better measure for this situation. New methods might be needed to accommodate the specific features of the sequencing data.

When pinpointing specific genes, except *FLT1*, the empirical power of all three methods was low. For *FLT1* there were eight rare and three common causal variants; the effect sizes of the common variants ranged from 0.61 to 0.74. In contrast, the power to detect *KDR*, which has eight rare and two common variants and roughly the same genetic profile as *FLT1*, was very low. A possible explanation could be that the amount of information contained in the two genes is different. Here, the EB estimates at the gene level could be a useful tool; in Figure 2 the median value of *FLT1* was higher than that of *KDR*. In addition, when common and rare variants are analyzed together, the common variants might play a significant role. The effect sizes of the common variants in *FLT1* (0.61–0.74) were larger that those in *KDR* (0.14 and 0.30).

We investigated the use of pathway analysis methods by jointly considering common and rare variants for sequencing data. We report promising performance of the WGT, which gives larger weights to rare variants compared to other methods intended for detecting common variants. Our conclusion is that straightforward application of pathway analysis is not satisfactory; hence, when common and rare variants are jointly analyzed using sequencing data, larger weights should be assigned to rare variants.

## Competing interests

The authors declare that there are no competing interests.

## Authors’ contributions

HWU preprocessed data, performed the analyses, and wrote the manuscript. HWU and JJHD participated in the development of the methods. HWU, RT, and JJHD interpreted the results of the analysis. All authors read and approved the final manuscript.
